# Supplementary material for: Evolution of Chlorhexidine Susceptibility and of the EfrEF Operon among Enterococcus faecalis from Diverse Environments, Clones, and Time Spans
Source: Microbiol Spectr. 2022 Jul 7;10(4):e01176-22. doi: 10.1128/spectrum.01176-22 (PMC9430118; doi:10.1128/spectrum.01176-22)
Supplement: Supplemental file 1 — Supplemental material. Download spectrum.01176-22-s0001.pdf, PDF file, 0.9 MB [file spectrum.01176-22-s0001.pdf]

Table S1 – Epidemiological background of the 181 *E. faecalis* included in the CHX susceptibility phenotypic assays.

Origin (n isolates)	Source (n isolates)	Date	Country (n isolates)	ST (n isolates)	CT (n isolates)	MDR
HUMANS n=101	Human infection: diverse biological products (47)	1996-2019	Portugal (43), Tunisia (4)	2 (1), 6 (10), 9 (2), 16 (3), 21 (2), 22 (1), 25 (1), 30 (1), 40 (4), 41 (1), 55 (2), 59 (1), 64 (1), 97 (1), 116 (1), 126 (1), 133 (1), 159 (1), 191 (1), 200 (2), 275 (1), 286 (1), 319 (1), 397 (1), 631 (1), 679 (1), 1105 (1), 1164 (1), 1165 (1)	1248 (3), 1252 (1), 1255 (1), 1265 (1), 1266 (1), 1267 (1), 1271 (1), 1376 (1), 1387 (1), 1393 (1), 1408 (1), 1412 (1), 1421 (1), 1422 (1), 1427 (1), 1428 (1), 1431 (1), 1435 (1), 1438 (1), 1439 (1), 1442 (1), 1448 (1), 1460 (1), 1463 (1), 1464 (1), 1471 (1), 1473 (1), 1474 (1), 1475 (1), 1479 (1), 1481 (1), 1486 (1), 1487 (1), 1647 (1), 2904 (1), 2905 (1), 2906 (1), 2907 (1), NA (7)	47%
	Healthy humans colonization: faeces (27), urinary tract (14), vagina (2), breast milk (1)	2001-2018	Portugal (42), Angola (2)	16 (1), 21 (1), 23 (1), 30 (1), 40 (20), 55 (2), 56 (1), 63 (1), 64 (1), 81 (2), 96 (1), 116 (1), 117 (1), 168 (1), 179 (2), 191 (1), 200 (1), 206 (1), 209 (1), 275 (1), 308 (1), 394 (1)	1215 (2), 1216 (1), 1218 (1), 1219 (1), 1228 (4), 1229 (1), 1232 (2), 1293 (1), 1294 (1), 1295 (1), 1309 (1), 1311 (1), 1319 (1), 1320 (1), 1321 (1), 1323 (1), 1325 (2), 1326 (2), 1328 (1), 1334 (1), 1337 (1), 1340 (1), 1343 (1), 1344 (1), 1346 (1), 1348 (1), 1350 (1), 1352 (1), 1356 (1), 1380 (1), 1489 (1), 1491 (1), 1493 (1), 1503 (2), 1567 (1), 1665 (1)	43%
	Long-term care patients: faeces (7)	2015-2016	Portugal (7)	25 (1), 26 (1), 40(1), 143 (1), 191 (1), 398 (1), 679 (1)	1359 (1), 1364 (1), 1365 (1), 1366 (1), 1368 (1), 1372 (1), 1645 (1)	14%
	Patients at hospital admission: faeces (3)	2015-2016	Brasil (3)	6 (2), 525 (1)	1296 (2), 1298 (1)	100%
FOOD CHAIN n=65	Animal production settings: aquaculture (11), piggery (9), poultry and ovine faeces (3)	2006-2015	Portugal (20), Tunisia (3)	21 (1), 22 (2), 35 (1), 40 (6), 59 (1), 65 (1), 100 (1), 139 (1), 200 (1), 209 (1), 330 (2), 386 (1), 445 (1), 631 (1), 749 (1), 872 (1)	1236 (1), 1246 (1), 1262 (1), 1439 (1), 1505 (1), 1506 (1), 1511 (1), 1513 (1), 1519 (1), 1520 (1), 1524 (1), 1528 (1), 1534 (1), 1537 (2), 1542 (1), 1543 (1), 1545 (1), 1546 (1), 1548 (1), 1549 (1), 1551 (1), 1556 (1)	39%
	Food of animal origin: poultry carcass (13), trout (8), bovine and goat milk (6), bovine meat (3), raw meat frozen pet food (6)	1999-2020	Portugal (25), Tunisia (11)	4 (1), 16 (2), 21 (1), 22 (1), 25 (1), 27 (1), 30 (1), 32 (1), 34 (1), 40 (2), 49 (1), 68 (1), 82 (1), 117 (1), 141 (1), 200 (1), 202 (1), 206 (1), 209 (1), 227 (1), 249 (1), 288 (1), 436 (1), 648 (1), 674 (1), 721 (1), 843 (1), 860 (1), 1106 (1), 1107 (1), 1008 (3), 1009 (1)	1205 (2), 1206 (1), 1207 (1), 1208 (1), 1209 (1), 1235 (1), 1238 (1), 1240 (1), 1242 (1), 1243 (1), 1269 (1), 1275 (1), 1285 (1), 1288 (1), 1290 (1), 1292 (1), 1494 (1), 1557 (1), 1558 (1), 1559 (1), 1560 (1), 1561 (1), 1563 (1), 1564 (1), 1565 (1), 1569 (1), 1571 (1), 1576 (1), 1583 (1), 1588 (1), 1589 (1), 1600 (1), 1625 (1), 1629 (1), 1640 (1)	39%
	Ready-to-eat salads (6)	2010	Portugal (6)	100 (1), 141 (1), 165 (1), 309 (1), 579 (1), 594 (1)	1496 (1), 1497 (1), 1498 (1), 1499 (1), 1500 (1), 1501 (1)	0%
PET FAECES n=3	Pet faeces: dog (1), cat (1), bird (1)	2014-2015	Tunisia (3)	21 (2), 116 (1)	1258 (1), 1260 (1), 1264 (1)	33%
AQUATIC ENVIRONMENT n=12	River (2)	2003	Portugal (2)	1 (1), 4 (1)	1 (1), 1467 (1)	0%
	Hospital sewage (6)	2001-2002	Portugal (6)	6 (1), 16 (1), 21 (1), 35 (1), 49 (1), 206 (1)	1651 (1), 1653 (1), 1656 (1), 1658 (1), 1659 (1), 1661 (1)	67%
	Urban wastewater treatment plant (4)	2014-2015	Tunisia (4)	4 (1), 23 (1), 86 (1), 117 (1)	20 (1), 1238 (1), 1241 (1), 1244 (1)	25%

Abbreviations: ST, sequence type; CT, complex type; MDR, multidrug resistance; NA, not available.

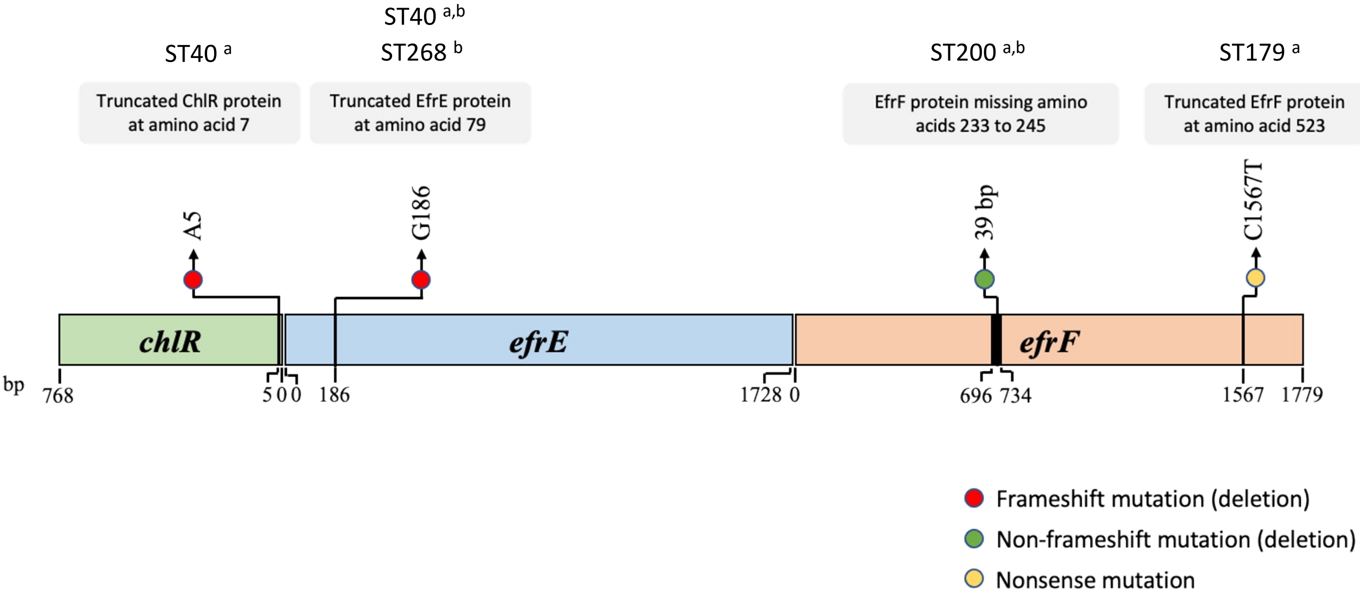

Fig S1. Gene mutations in *chlR* (green), *efrE* (blue) and *efrF* (orange) resulting in incomplete proteins detected among the 666 sequenced *E. faecalis* genomes from our collection[a] or published at the PATRIC database[b] (when protein sequences had 100% identity until the stop codon with those found in our isolates with incomplete ChlR-EfrEF). The sequence type (ST) of isolates in which the mutations were found are indicated. Frameshift, non-frameshift or nonsense mutations are marked with different colours. Detailed information on the epidemiological background and chlorhexidine phenotypes of isolates from our collection carrying incomplete ChlR-EfrEF proteins is available on Fig. 3 and Table S2.

TABLE S2 – Distribution of the ChlR-EfrEF variants among *Enterococcus faecalis* isolates showing different chlorhexidine phenotypes and with diverse epidemiological backgrounds.

Strain	MIC (mg/L)	MBC (mg/L)	ChlR-EfrEF mutations [1] ChlR	EfrE	EfrF	Source	Geographical Region	Date	ST	CT
C299	0.5	8	L185V	Tr [b]	R58H; P67S; E365K	HH	Portugal	2001	40	1325
c20Ua_66_AE	0.5	8	S188P	Q234R; K435E	I [c]	HH	Portugal	2017	200	1320
TR 52-42	0.5	32	S188P	Q234R; K435E	I [c]	T	Portugal	2012	200	1558
H189	1	8	Tr [a]	-	-	HI	Portugal	2002	319	1422
C72	1	1	L185V	Tr [b]	R58H; P67S; E365K	HH	Portugal	2001	40	1226
C287	1	2	L185V	Tr [b]	R58H; P67S; E365K	HH	Portugal	2001	40	1328
C273	1	4	L185V	Tr [b]	R58H; P67S; E365K	HH	Portugal	2001	40	1325
C325	1	4	L185V	Tr [b]	R58H; P67S; E365K	HH	Portugal	2001	40	1334
c4Ua_1_AE	1	4	L185V	Tr [b]	R58H; P67S; E365K	HH	Portugal	2016	40	1219
c7Ua_50_AE	1	4	L185V	Tr [b]	R58H; P67S; E365K	HH	Portugal	2017	40	1228
c7ua_173_AE	1	4	L185V	Tr [b]	R58H; P67S; E365K	HH	Portugal	2017	40	1228
c9VSa_38_AE	1	4	L185V	Tr [b]	R58H; P67S; E365K	HH	Portugal	2017	40	1232
GE18C1	1	8	L185V	Tr [b]	R58H; P67S; E365K	HH	Portugal	2001	40	1665
C27	1	8	L185V	Tr [b]	R58H; P67S; E365K	HH	Portugal	2001	40	1326
C452	1	8	L185V	Tr [b]	R58H; P67S; E365K	HH	Portugal	2001	40	1346
TR 32-23	1	8	L185V	Tr [b]	R58H; P67S; E365K	T	Portugal	2011	40	1545
TR 32-28	1	8	L185V	Tr [b]	R58H; P67S; E365K	T	Portugal	2011	40	1546
c9Ua_177_AE	1	8	L185V	Tr [b]	R58H; P67S; E365K	HH	Portugal	2017	40	1232
H218	1	16	L185V	Tr [b]	R58H; P67S; E365K	HI	Portugal	2002	40	1439
TR 58-30	1	16	L185V	Tr [b]	R58H; P67S; E365K	T	Portugal	2012	40	1560
c2Ua_11_AN	1	16	L185V	Tr [b]	R58H; P67S; E365K	HH	Portugal	2016	40	1215
c7Vsa_42_AE	1	16	L185V	Tr [b]	R58H; P67S; E365K	HH	Portugal	2018	40	1228
C355	1	32	L185V	Tr [b]	R58H; P67S; E365K	HH	Portugal	2001	40	1337
TR 17_48	1	32	L185V	Tr [b]	R58H; P67S; E365K	T	Portugal	2011	40	1439
c3Ua_16_AE	1	32	L185V	Tr [b]	R58H; P67S; E365K	HH	Portugal	2016	40	1215
PF63	1	64	L185V	Tr [b]	R58H; P67S; E365K	PF	Portugal	2019	40	1206
H63	1	32	S188P	Q234R; K435E	I [c]	HI	Portugal	2001	200	1463
H161	1	32	S188P	Q234R; K435E	I [c]	HI	Portugal	2002	200	1412
SN48	1	32	S188P	Q234R; K435E	I [c]	P	Portugal	2007	200	1524
c24ua_119_AE	1	8	D138N	V264I; A369S	Tr [d]	HH	Portugal	2018	179	1323
C433	1	16	S188P	Q234R; T324I; S470A; S519A	D240A; V372I; G493R	HH	Portugal	2001	308	1343
CCM27	1	64	S188P	Q234R; T324I; S470A; S519A	D240A; V372I; G493R	LTCP	Portugal	2016	40	1365
c10Ua_167_AE	2	4	L185V	Tr [b]	R58H; P67S; E365K	HH	Portugal	2001	30	1348
692T	2	16	NF	NF	NF	PC	Tunisia	2017	860	1288
SE93C4	2	8	-	-	-	HH	Portugal	2001	55	1503
376T	2	8	-	-	-	HI	Portugal	2016	55	1271
F1191	2	32	-	A63T; Q234R; A369S	-	PC	Portugal	2018	202	1583
E44	2	8	-	Q234R; A369S	-	HS	Portugal	2001	21	1661
HPH25	2	16	-	Q234R; E386D	L297S; V329F	HI	Portugal	2019	191	2905
C504	2	8	-	A369S	S137L; A207S	HH	Portugal	2001	168	1350
C469	2	4	-	A369S	A207S; E567K	HH	Portugal	2017	40	1228
137T	2	8	S188P	Q234R; T324I; S470A; A501T; S519A	D240A	BGM	Portugal	2015	25	1242
H153	4	8	Tr [a]	N11S	L7I; R58H; P67S; D240A	HI	Portugal	2001	59	1408
H99	4	16	L185V	Tr [b]	R58H; P67S; E365K	HI	Portugal	2001	40	1473
CHCB4	4	4	-	-	-	HI	Portugal	2007	6	1376
229710	4	8	-	-	-	HI	Portugal	1996	6	1255
H65	4	8	-	-	-	HI	Portugal	2001	6	1464
H209	4	8	-	-	-	HI	Portugal	2002	55	1435
VE79C1	4	8	-	-	-	HH	Portugal	2001	55	1503
M110748	4	8	-	-	-	HI	Portugal	2010	6	1248
Br54_1	4	8	-	-	-	HH	Brasil	2016	6	1296
M209565	4	16	-	-	-	HI	Portugal	2011	6	1248
TR39_20	4	16	-	-	-	AQ	Portugal	2011	139	1549
M149277	4	32	-	-	-	HI	Portugal	2010	6	1248
158T	4	32	-	-	-	AF	Tunisia	2015	65	1246
698T	4	32	-	-	-	BM	Tunisia	2017	1107	1290
F1155	4	16	-	-	L7I; R58H; P67S	PC	Portugal	2018	843	1571
TR55-3	4	32	-	-	D240A; K419E	T	Portugal	2012	227	1559
F805	4	16	-	-	A577T	PC	Portugal	2001	648	1625
SN453	4	16	-	T21A; Q234R; A369S	-	P	Portugal	2007	21	1520
515T	4	8	-	T21A; Q234R; E386D	-	BM	Tunisia	2016	21	1275
VE5C2	4	16	-	T21A; A369S	-	HH	Portugal	2001	21	1567
SN163	4	8	-	A63T; Q234R; A369S	-	P	Portugal	2007	22	1505
H215	4	16	-	A63T; Q234R; A369S	-	HH	Portugal	2002	22	1438
F186	4	32	-	A63T; Q234R; A369S	-	PC	Portugal	1999	82	1588
237T	4	16	-	A63T; Q234R; E386D	-	AF	Tunisia	2015	22	1236
F1023	4	16	-	A63T; Q234R; E386D	-	PC	Portugal	2018	22	1569
E290	4	16	-	Q234R; I254L; D520E	L7I; R58H; D240A; I277T; L278F	HS	Portugal	2002	35	1659
SN203	4	32	-	Q234R; I254L; D520E	L7I; R58H; D240A; I277T; L278F	P	Portugal	2008	35	1506

TABLE S2 (continued) - Distribution of the ChlR-EfrEF variants among *Enterococcus faecalis* isolates showing different chlorhexidine phenotypes and with diverse epidemiological backgrounds.

Strain	MIC (mg/L)	MBC (mg/L)	ChlR-EfrEF mutations [1] ChlR	EfrE	EfrF	Source	Geographical Region	Date	ST	CT
Med88C2	4	16	-	Q234R; V357F; Q409K	D240A	HH	Portugal	2001	209	1493
SN790	4	16	-	Q234R; V357F; Q409K	D240A	P	Portugal	2007	209	1534
99T	4	32	-	Q234R; V357F; Q409K	D240A	BGM	Tunisia	2014	209	1292
CHCB5	4	16	-	Q234R; A369S	-	HI	Portugal	2008	21	1647
290T	4	32	-	Q234R; A369S	-	F	Tunisia	2015	21	1258
CCT29	4	16	-	Q234R; A369S	L297S; V329F	LTCP	Portugal	2016	191	1359
c13Ua_6_AN	4	32	-	Q234R; A369S	L297S; V329F	HH	Portugal	2017	191	1309
TR9_12	4	32	-	Q234R; A369S; V410I; S519A	L74F	AQ	Portugal	2010	330	1537
TR10_30	4	32	-	Q234R; A369S; V410I; S519A	L74F	AQ	Portugal	2010	330	1537
H109	4	4	-	Q234R; E386D	-	HI	Portugal	2001	21	1387
307T	4	16	-	Q234R; E386D	-	F	Tunisia	2014	21	1260
F1109	4	4	-	Q234R; Q409K	-	PC	Portugal	2018	16	1576
R8	4	16	-	Q234R; Q409K	-	R	Portugal	2003	4	1463
614T	4	16	-	Q234R; Q409K	-	PC	Tunisia	2017	16	1285
TR76-27	4	32	-	Q234R; Q409K	D240A; A577T	T	Portugal	2012	436	1565
F534	4	8	-	Q234R; Q409K	M400I	PC	Portugal	2001	32	1600
E274	4	16	-	V264I; A369S	-	HS	Portugal	2002	49	1658
S37_25E	4	32	-	V264I; E386D	-	RS	Portugal	2010	309	1499
H92	4	16	-	E330K; Q409K	P67S	HI	Portugal	2001	631	1471
SN60	4	32	-	E330K; Q409K	P67S	P	Portugal	2007	631	1528
S36_24E	4	32	-	N353S; A369S; K435E	-	RS	Portugal	2010	141	1498
TR52_41	4	32	-	N353S; E386D; K435E	-	T	Portugal	2012	141	1557
HVR7	4	4	-	A369S	A207S; E567K	HI	Portugal	2007	30	1486
c21Ua_42_M	4	8	-	A369S	A207S; E567K	HH	Portugal	2017	56	1321
SN38	4	32	-	A369S; K435E; S519A	P67S	P	Portugal	2007	100	1519
104T	4	4	-	E386D	A207S; E567K	BGM	Tunisia	2014	30	1235
S52_30E	4	32	-	E386D; K435E; S519A	P67S	RS	Portugal	2010	100	1501
F23	4	8	-	K435E	-	PC	Portugal	1999	27	1589
CCT70	4	8	-	K435E	L278F	LTCP	Portugal	2016	26	1645
204070	4	8	-	S519A	P67S	HI	Portugal	1999	159	1252
H197	4	4	-	A554D	L7I; R58H; P67S	HI	Portugal	2002	275	1427
C20	4	16	-	A554D	L7I; R58H; P67S	HH	Portugal	2001	275	1319
SN369	4	32	E86K; D138N	Q234R; A369T; Q409K	-	P	Portugal	2008	445	1511
KE10C1	4	16	H98Q	Q234R; V354A; K435E	-	HH	Portugal	2001	206	1489
131T	4	8	H98Q	Q234R; K435E	-	BGM	Tunisia	2015	206	1240
C418	4	4	H98Q; E222K	Q234R; K435E	-	HH	Portugal	2001	23	1344
14T	4	16	H98Q; E222K	Q234R; K435E	-	UWTP	Tunisia	2014	23	1241
E204	4	4	H98Q; E254K	Q234R; K435E	-	HS	Portugal	2001	206	1651
TR59_34	4	32	A112V	E386D	-	T	Portugal	2012	68	1561
A21_1A	4	4	V120F	Q268E; A369S	D240A	HH	Angola	2015	116	1293
HVR18	4	8	V120F	Q268E; A369S	D240A	HI	Portugal	2008	116	1481
300T	4	8	V120F	Q268E; A369S	D240A	F	Tunisia	2015	116	1264
501T	4	16	D138N	P33S; V264I; A369S	-	HI	Tunisia	2014	64	1265
BM8	4	32	D138N	P33S; V264I; A369S	-	HH	Portugal	2017	64	1295
P619	4	32	D138N	Q234R; S519A	-	PC	Portugal	2001	249	1494
C584	4	4	D138N	V264I; A369S	-	HH	Portugal	2001	117	1356
CCM79	4	8	D138N	V264I; A369S	-	LTCP	Portugal	2016	679	1368
13T	4	16	D138N	V264I; A369S	-	UWTP	Tunisia	2014	117	1244
138T	4	16	D138N	V264I; A369S	-	BGM	Tunisia	2015	117	1243
c14Uc_159_AE	4	16	D138N	V264I; A369S; A554D	-	HH	Portugal	2018	179	1311
TR49_34	4	32	L185F	F96I; A369S	P67S; E365K	AQ	Portugal	2012	749	1556
R6	4	16	L185V	Q234R	A207S	R	Portugal	2003	1	1
TR35-29	4	32	L185V	Q234R; K435E; S519A	G60E	AQ	Portugal	2011	386	1548
C546	4	8	L185V	K435E; A516T; A554D	L7I; R58H; P67S; H246Q; E365K	HH	Portugal	2001	96	1352
360T	4	8	S188P	-	L278F	BM	Tunisia	2016	34	1269
S40_40E	4	32	S188P	-	L278F	RS	Portugal	2010	579	1500
497T	4	4	S188P	Q234R; T324I; S470A; S519A	D240A	HI	Tunisia	2011	133	1267
393T	4	8	S188P	Q234R; T324I; S470A; S519A	D240A	HI	Tunisia	2015	25	1266
H6	4	16	S188P	Q234R; T324I; S470A; S519A	D240A	HI	Portugal	2001	41	1460
H45	4	16	S188P	Q234R; T324I; S470A; S519A	D240A	HI	Portugal	2001	97	1448
CCM48	4	16	S188P	Q234R; T324I; S470A; S519A	D240A	LTCP	Portugal	2016	25	1366
273T	4	32	S188P	Q234R; T324I; S470A; S519A	D240A	AF	Tunisia	2015	872	1262
S32_12E	4	16	S188P	Q234R; M431T; K435E; S470A; S519A; L524F	D240A	RS	Portugal	2010	594	1497
PF56	4	16	S188P	Q234R; S470A; S519A	D240A; A577T	PF	Portugal	2019	1008	1205
PF101	4	32	S188P	Q234R; S470A; S519A	D240A; A577T	PF	Portugal	2019	1008	1209
S31_4E	4	8	S188P	Q234R; S519A	D240A	RS	Portugal	2010	165	1496
TR68_32	4	8	S188P	H320N	D240A; L278F	T	Portugal	2012	1106	1564
KE44C1	4	8	S188P; E211K	K231I; E386D; K435E; S519A	N51K; P67S	HH	Portugal	2001	81	1491
A35_1A	4	8	S188P; E211K	A369S; K435E; S519A	N51K; P67S; P578Q	HH	Angola	2013	81	1294
F871	4	8	S188P; I242V	-	D240A	PC	Portugal	2001	288	1629
H118	4	8	E211G	Q234R; K435E	R58H; P67S; E365K	HI	Portugal	2001	9	1393
69T	4	16	V226I	Q234R; Q409K; L557I	-	UWTP	Tunisia	2015	86	20
CCM22	4	32	I249V	-	L278F	LTCP	Portugal	2016	143	1364
PF110	4	32	I249V	M116I; V300I	L278F	PF	Portugal	2019	674	1207

TABLE S2 (continued) - Distribution of the ChlR-EfrEF variants among *Enterococcus faecalis* isolates showing different chlorhexidine phenotypes and with diverse epidemiological backgrounds.

Strain	MIC (mg/L)	MBC (mg/L)	ChlR-EfrEF mutations [1]			Source	Geographical Region	Date	ST	CT
			ChlR	EfrE	EfrF					
E243	8	8	-	-	-	HS	Portugal	2002	6	1653
HVR2	8	8	-	-	-	HI	Portugal	2007	6	1479
Br02_1	8	8	-	-	-	HS	Portugal	2015	6	1296
HPH7	8	16	-	-	-	HI	Portugal	2007	6	1474
M5366845	8	16	-	-	-	HI	Portugal	2013	6	2907
CCT120	8	32	-	-	Q233P; E236K	LTCP	Portugal	2016	398	1372
PF183	8	16	-	-	D240A	PF	Portugal	2020	1009	1208
SN289	8	16	-	N11S	L7I; R58H; P67S; D240A	P	Portugal	2007	59	1513
HPH20	8	32	-	N11S	L7I; R58H; P67S; D240A	HI	Portugal	2019	1105	2904
C370	8	8	-	Q234R	D240A	HH	Portugal	2001	63	1340
E263	8	8	-	Q234R; Q409K	-	HS	Portugal	2002	16	1653
HVR10	8	8	-	Q234R; Q409K	-	HI	Portugal	2008	16	1475
c3Ub_209_AE	8	16	-	Q234R; Q409K	-	HH	Portugal	2017	16	1216
HPH32	8	32	-	Q234R; Q409K	-	HI	Portugal	2019	16	2906
F916	8	32	-	Q234R; K435E; S519A; T552S	P67S	PC	Portugal	2001	721	1640
TR63_37	8	16	-	V264I; A369S	-	T	Portugal	2012	49	1563
11T	8	16	-	N353S; Q409K	-	UWTP	Tunisia	2014	4	1238
127T	8	16	-	N353S; Q409K	-	BGM	Tunisia	2015	4	1238
GE7C1	8	32	-	A369S	-	HH	Portugal	2001	394	1380
Br16	8	16	-	S519A	P67S	HH	Portugal	2015	525	1298
HVR8	8	32	D138N	V264I; A369S	S347F	HI	Portugal	2007	397	1486
H201	8	8	Y145C	-	T188N	HI	Portugal	2002	2	1431
c8Ua_2_AE	8	16	L185V	Q234R; K435E	R58H; P67S; E365K	HH	Portugal	2017	40	1229
H188	8	32	L185V	Q234R; K435E	R58H; P67S; E365K	HI	Portugal	2002	40	1421
H198	8	32	L185V	Q234R; K435E	R58H; P67S; E365K	HI	Portugal	2002	40	1428
TR31_26	8	32	L185V	Q234R; K435E	R58H; P67S; E365K	AQ	Portugal	2011	40	1543
TR37_41	8	32	L185V	Q234R; K435E	R58H; P67S; E365K	AQ	Portugal	2011	40	1551
C47	8	64	L185V	Q234R; K435E	R58H; P67S; E365K	HH	Portugal	2001	40	1218
TR21_60	8	64	L185V	Q234R; K435E	R58H; P67S; E365K	AQ	Portugal	2011	40	1542
PF176	8	16	S188P	Q234R; S470A; S519A	D240A; A577T	PF	Portugal	2020	1008	1205
H307	8	8	E211G	S46G; Q234R; K435E	R58H; P67S; E365K	HI	Portugal	2002	9	1442

[1] Amino acid variation.

[a] Truncated ChlR at amino acid 7. [b] Truncated EfrE at amino acid 79. [c] Incomplete EfrF missing amino acids 233 to 245. [d] Truncated EfrF at amino acid 523. The reference strain used was *E. faecalis* V583 [GenBank accession no. AE016830.1; locus-tag EF_2225 (*chlR*), EF_2226 (*efrE*) and EF_2227 (*efrF*)].

Abbreviations: -, no mutations detected; AF, animal faeces; AQ, aquaculture; BGM, bovine and goat milk; BM, bovine meat; F, pet faeces; HA, human faecal samples in hospital admission; HH, healthy-humans; HI, human infection; HS, hospital sewage; I, incomplete; LTCP, long-term care patients; MIC, minimum inhibitory concentration; MBC, minimum bactericidal concentration; NA, not available; NF, not found; P, piggery; PC, poultry carcass; PF, raw frozen pet food; T, trout; Tr, truncated ChlR, EfrE or EfrF protein; R, river water; RS, ready-to-eat salads; ST, sequence type; UWTP, urban wastewater treatment plant.

TABLE S3 – ChlR-EfrEF variability and epidemiological background of *E. faecalis* genomes identified as ST40 from our collection and available at the PATRIC database (until the 18th of December 2020) (n=122).

ChlR-EfrEF proteins			*E. faecalis* strain	Date	Isolation Source	Geographic Location	Genome ID PATRIC	GenBank Acc. / Bioproject Number
ChlR	EfrE	EfrF						
+	Tr [a]	+	NCTC8175	1900-1950	Milk	UK	1351.1221	UGIM01000000
			E1	1960s	Human infection	USA	1158673.3	AJEU00000000
			T15	1973	Human infection	USA	1158672.3	AJET00000000
			JH1	<1975	Human infection	UK	565648.4	ACAP00000000
			DS16	1978	Human infection	USA	1158677.3	AJEY00000000
			RC73	1979	Human infection	USA	1158678.3	AJEZ00000000
			A-3-1	early 1980s	Human infection	USA	1169246.3	ASEF00000000
			B289	1984	Human	USA	1391472.3	JAHH00000000
			B292	1984	Human	USA	1391474.3	JAHJ00000000
			B316	1984	Human	USA	1391480.3	JAHP00000000
			B319	1984	Human	USA	1391482.3	JAHR00000000
			B363	1984	Human	USA	1391493.3	JAIC00000000
			B291	1985	Human	USA	1391473.3	JAHI00000000
			B293	1985	Human	USA	1391475.3	JAHK00000000
			B294	1985	Human	USA	1391476.3	JAHL00000000
			B302	1985	Human	USA	1391478.3	JAHN00000000
			B324	1985	Human	USA	1391485.3	JAHU00000000
			B338	1985	Human	USA	1391488.3	JAHX00000000
			B348	1985	Human	USA	1391491.3	JAIA00000000
			GE18C1	2001	Human colonization	Portugal		PRJEB28327
			C27	2001	Human colonization	Portugal		PRJEB28327
			C72	2001	Human colonization	Portugal		PRJEB28327
			C273	2001	Human colonization	Portugal		PRJEB28327
			C287	2001	Human colonization	Portugal		PRJEB28327
			C299	2001	Human colonization	Portugal		PRJEB28327
			C325	2001	Human colonization	Portugal		GCA_905123595.1
			C355	2001	Human colonization	Portugal		PRJEB28327
			C452	2001	Human colonization	Portugal		PRJEB28327
			H99	2001	Human infection	Portugal		PRJEB28327
			H218	2002	Human infection	Portugal		PRJEB28327
			Merz192	2002	Human infection	USA	1169248.3	ASEH00000000
			Merz204	2002	Human infection	USA	1169249.3	ASEI00000000
			Merz89	2002	Human infection	USA	1169250.3	ASEG00000000
			12	2011	Copper fed pig	Denmark	1351.116	JTKT00000000
			TR17_48	2011	Aquaculture	Portugal		PRJEB28327
			TR32_23	2011	Aquaculture	Portugal		PRJEB28327
			TR32_28	2011	Aquaculture	Portugal		PRJEB28327
			TR58_30	2012	Trout	Portugal		PRJEB28327
			1187_EFLS	2012-2013	Human infection	USA	1351.233	JVZS01000000
			258.rep2_EFLS	2012-2013	Human infection	USA	1351.242	JVOA01000000
			391_EFLS	2012-2013	Human infection	USA	1351.244	JVIY01000000
			406_EFLS	2012-2013	Human infection	USA	1351.245	JVIK01000000
			581_EFLS	2012-2013	Human infection	USA	1351.248	JVBG01000000
			213_EFLS	2012-2013	Human infection	USA	1351.255	JVPS01000000
			257_EFLS	2012-2013	Human infection	USA	1351.259	JVOC01000000
			388_EFLS	2012-2013	Human infection	USA	1351.262	JVJB01000000
			601_EFLS	2012-2013	Human infection	USA	1351.270	JVAM01000000
			621_EFLS	2012-2013	Human infection	USA	1351.271	JUZT01000000
			703_EFLS	2012-2013	Human infection	USA	1351.276	JUWK01000000
			948_EFLS	2012-2013	Human infection	USA	1351.289	JUMK01000000
			133170041-3	2013	Air	USA: International Space Station	1351.3790	GCA_013344605.1
			HUM-328	2013	Human colonization	Estonia	1351.4120	JACZBA000000000
			UM001B	2013	Human infection	USA	1351.4157	CP053026.1
			0519A_37_406	2013/2014	Human colonization	USA	1351.440	MKXV00000000
			ICU1-2c	2014	NA	USA	1351.328	LQAM00000000
			H114S2	2014	Wastewater (primary effluent)	Canada	1351.3401	WMGT01000000
			W195	2015	Wastewater (final effluent)	Canada	1351.3409	WMGS01000000
			R395	2015	Wastewater (primary effluent)	Canada	1351.3411	WMGP01000000
			C379	2016	Wastewater (final effluent)	Canada	1351.3404	WMGU01000000
			c2Ua_11_AN	2016	Human colonization	Portugal		PRJEB28327
			c3Ua_16_AE	2016	Human colonization	Portugal		PRJEB28327
			c4Ua_1_AE	2016	Human colonization	Portugal		PRJEB28327
			L3894	2017	Human colonization	USA	1351.3473	WVTT01000000
			E3894	2017	Human colonization	USA	1351.3475	WVTL01000001
			VRE 59	2017	NA	Lebanon	1351.4086	JAAIGK010000000

TABLE S3 (continued) – ChlR-EfrEF variability and epidemiological background of *E. faecalis* genomes identified as ST40 from our collection and available at the PATRIC database (until the 18th of December 2020) (n=122).

ChlR-EfrEF proteins			*E. faecalis* strain	Date	Isolation Source	Geographic Location	Genome ID PATRIC	GenBank Acc. / Bioproject Number
ChlR	EfrE	EfrF						
+	Tr [a]	+	c7Ua_50_AE	2017	Human colonization	Portugal		GCA_905123655.1
			c7ua_173_AE	2017	Human colonization	Portugal		PRJEB28327
			c9Ua_177_AE	2017	Human colonization	Portugal		PRJEB28327
			c10Ua_167_AE	2017	Human colonization	Portugal		PRJEB28327
			c7Vsa_42_AE	2018	Human colonization	Portugal		PRJEB28327
			c9VSa_38_AE	2018	Human colonization	Portugal		PRJEB28327
			4928STDY7071218	2018	Human colonization	UK	1351.2101	CABGHW000000000
			4928STDY7071474	2018	Human colonization	UK	1351.2120	CABGJI000000000
			4928STDY7071574	2018	Human colonization	UK	1351.2124	CABGJT000000000
			4928STDY7071435	2018	Human colonization	UK	1351.2169	CABGOB000000000
			4928STDY7071473	2018	Human colonization	UK	1351.2180	CABGPI000000000
			4928STDY7071472	2018	Human colonization	UK	1351.2185	CABGPH000000000
			4928STDY7071537	2018	Human colonization	UK	1351.2198	CABGRJ000000000
			4928STDY7071576	2018	Human colonization	UK	1351.2207	CABGSN000000000
			4928STDY7071575	2018	Human colonization	UK	1351.2210	CABGSG000000000
			4928STDY7071281	2018	Human colonization	UK	1351.2272	CABGYG000000000
			4928STDY7071723	2018	Human colonization	UK	1351.2284	CABGZY000000000
			4928STDY7071736	2018	Human colonization	UK	1351.2298	CABHAI000000000
			4928STDY7071738	2018	Human colonization	UK	1351.2306	CABHAQ000000000
			4928STDY7071753	2018	Human colonization	UK	1351.2308	CABHBB000000000
			4928STDY7071754	2018	Human colonization	UK	1351.2317	CABHAW000000000
			4928STDY7071769	2018	Human colonization	UK	1351.2326	CABHBQ000000000
			4928STDY7387716	2018	Human colonization	UK	1351.2350	CABHDL000000000
			4928STDY7387750	2018	Human colonization	UK	1351.2364	CABHEG000000000
			4928STDY7387749	2018	Human colonization	UK	1351.2365	CABHEI000000000
			4928STDY7071263	2018	Human colonization	UK	1351.2431	LR607346
			BL-380-WT-3A	2018	Human colonization	Germany	1351.3672	JABAFU000000000
			PF63	2019	Raw frozen pet food	Portugal		PRJEB28327
			N039.H-3	NA	Human colonization (infant)	USA	1351.2073	QYNC01000000
			44.1	NA	NA	Spain	1351.591	NSJR00000000
			13.1	NA	NA	Spain	1351.592	NSJS00000000
			UBA7859	NA	Metal/plastic	USA	1351.655	DLSP01000000
			PC1.1	NA	Human colonization	Australia	791166.14	ADKN01000000
NA [b]	Tr [a]	+	UBA4546	NA	New York City MTA subway	USA	1351.634	DGLK01000000
Tr [c]	Tr [a]	+	ATCC 27959	1975	Bovine mastitis	USA	1158676.3	AJEX00000000
+	Tr [a]	Tr [d]	RM3817	1960s	Human infection	USA	1158674.3	AJEV00000000
+	+	Tr [e]	ATCC 10100	<1949	NA	NA	1169312.3	ASEA00000000
+	+	+	NCTC2705	1900-1927	Udder of cows	Denmark	1351.1229	UGIW01000000
			ATCC 27275	<1963	NA	NA	1158675.3	AJEW00000000
			7330082-2	2001	Human colonization	Denmark	1151198.3	AIIW00000000
			D1	2001	Pig	Denmark	1169247.3	ASEJ00000000
			D32	2001	Pig feaces	Denmark	1206105.3	CP003726
			C47	2001	Human colonization	Portugal		PRJEB28327
			H188	2002	Human infection	Portugal		PRJEB28327
			H198	2002	Human infection	Portugal		PRJEB28327
			19	2011	Copper fed pig	Denmark	1351.119	JTKW00000000
			TR21_60	2011	Aquaculture	Portugal		PRJEB28327
			TR31_26	2011	Aquaculture	Portugal		GCA_905123745.1
			TR37_41	2011	Aquaculture	Portugal		PRJEB28327
			H4	2014	Beef packing plant and retail meat	Canada	1351.2050	VHRN01000000
			CVM N52755	2014	Ground turkey	USA	1351.873	PTYC01000000
			W97	2015	Beef packing plant and retail meat	Canada	1351.2036	VHQO01000000
			G149	2016	Beef packing plant and retail meat	Canada	1351.2032	VHRX01000000
			CCM27	2016	Human colonization	Portugal		PRJEB28327
			c8Ua_2_AE	2017	Human colonization	Portugal		PRJEB28327
			NBRC 12965	NA	Citrus juice	NA	1351.2018	BJMP01000000
			TX4248	NA	Human infection	USA	749495.4	AEBR00000000

[a] Truncated EfrE at amino acid 79. [b] ChlR mutations were not assessed due to sequencing error. [c] Truncated ChlR at amino acid 181. [d] Truncated EfrF at amino acid 392. [e] Truncated EfrF at amino acid 146. The reference strain used was *E. faecalis* V583 [GenBank accession no. AE016830.1; locus-tag EF_2225 (*chlR*), EF_2226 (*efrE*) and EF_2227 (*efrF*)]. Abbreviations: +, complete protein; Tr, truncated protein; NA, not available; UK, United Kingdom; USA, United States of America.
